# Asymmetries of the subthalamic activity in Parkinson’s disease: phase-amplitude coupling among local field potentials

**DOI:** 10.1093/braincomms/fcae201

**Published:** 2024-06-11

**Authors:** Tommaso Bocci, Rosanna Ferrara, Tommaso Albizzati, Alberto Averna, Matteo Guidetti, Sara Marceglia, Alberto Priori

**Affiliations:** ‘Aldo Ravelli’ Research Center for Neurotechnology and Experimental Neurotherapeutics, Department of Health Sciences, University of Milan, 20142 Milan, Italy; III Neurology Clinic, ASST-Santi Paolo e Carlo University Hospital, 20142 Milan, Italy; ‘Aldo Ravelli’ Research Center for Neurotechnology and Experimental Neurotherapeutics, Department of Health Sciences, University of Milan, 20142 Milan, Italy; Department of Engineering and Architecture, University of Trieste, Trieste, 34127 Friuli-Venezia Giulia, Italy; Department of Neurology, Bern University Hospital and University of Bern, 3010 Bern, Switzerland; ‘Aldo Ravelli’ Research Center for Neurotechnology and Experimental Neurotherapeutics, Department of Health Sciences, University of Milan, 20142 Milan, Italy; Department of Engineering and Architecture, University of Trieste, Trieste, 34127 Friuli-Venezia Giulia, Italy; Newronika S.r.l., 20093 Cologno Monzese, Italy; ‘Aldo Ravelli’ Research Center for Neurotechnology and Experimental Neurotherapeutics, Department of Health Sciences, University of Milan, 20142 Milan, Italy; III Neurology Clinic, ASST-Santi Paolo e Carlo University Hospital, 20142 Milan, Italy

**Keywords:** deep brain stimulation, local field potentials, Parkinson’s disease, phase-amplitude coupling, asymmetry

## Abstract

The role of brain asymmetries of dopaminergic neurons in motor symptoms of Parkinson’s disease is still undefined. Local field recordings from the subthalamic nucleus revealed some neurophysiological biomarkers of the disease: increased beta activity, increased low-frequency activity and high-frequency oscillations. Phase-amplitude coupling coordinates the timing of neuronal activity and allows determining the mechanism for communication within distinct regions of the brain. In this study, we discuss the use of phase-amplitude coupling to assess the differences between the two hemispheres in a cohort of 24 patients with Parkinson’s disease before and after levodopa administration. Subthalamic low- (12–20 Hz) and high-beta (20–30 Hz) oscillations were compared with low- (30–45 Hz), medium- (70–100 Hz) and high-frequency (260–360 Hz) bands. We found a significant beta-phase-amplitude coupling asymmetry between left and right and an opposite-side-dependent effect of the pharmacological treatment, which is associated with the reduction of motor symptoms. In particular, high coupling between high frequencies and high-beta oscillations was found during the OFF condition (*P* < 0.01) and a low coupling during the ON state (*P* < 0.0001) when the right subthalamus was assessed; exactly the opposite happened when the left subthalamus was considered in the analysis, showing a lower coupling between high frequencies and high-beta oscillations during the OFF condition (*P* < 0.01), followed by a higher one during the ON state (*P* < 0.01). Interestingly, these asymmetries are independent of the motor onset side, either left or right. These findings have important implications for neural signals that may be used to trigger adaptive deep brain stimulation in Parkinson’s and could provide more exhaustive insights into subthalamic dynamics.

## Teaser

In patients with advanced Parkinson’s disease, Bocci *et al*. investigated asymmetries in local field oscillations between the two subthalamic nuclei. They showed significant differences, which are independent of the motor onset side, before and after levodopa intake. These findings may support unilateral surgical approaches, at least in selected patients.

## Graphical Abstract

**Graphical Abstract:**
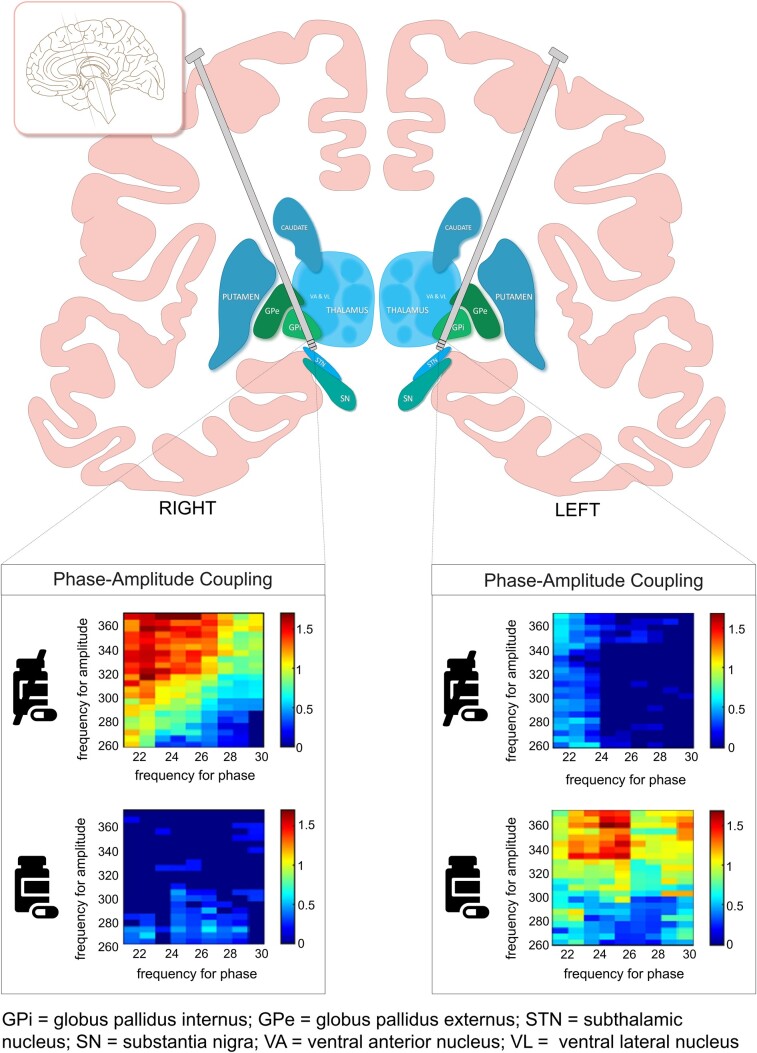

## Introduction

Parkinson’s disease (PD) is a neurodegenerative disorder characterized by the loss of dopaminergic cells in the *substantia nigra pars compacta* and other brainstem nuclei.^1^ Dopamine loss triggers a series of pathological changes in the basal ganglia, ultimately leading to the emergence of pathological dynamics.^2^ The emergence of exaggerated beta-band (11–30 Hz range) neuronal oscillations in the cortex and different basal ganglia nuclei, especially in the subthalamic nucleus (STN),^2^ is one of the abnormal functional changes following dopamine loss in Parkinson’s disease.^3^ However, the relationship between neuronal firing rate changes and local field potential (LFP)-recorded abnormal beta oscillations is still a matter of debate, probably depending on the functional changes occurring within the basal ganglia–cortical network.^4,5^ Two main, not mutually exclusive, hypotheses were proposed.^6^ First, these oscillations reflect the activity in an STN-globus pallidus externus loop that is generated after dopamine denervation; second, they may arise from the activation of the hyper-direct cortico-STN pathway.^7-10^ Beta-band pathological synchronous oscillatory activity, recorded through oscillatory beta LFPs, is now considered the most promising biomarker for controlling novel deep brain stimulation (DBS) approaches (adaptive DBS, aDBS).^11-16^

Indeed, beta oscillations strongly correlate with movement preparation and execution,^17^ akinesia^18^ and motor imagery,^19^ and the reduction of their activity by levodopa administration correlates with a clinical reduction in the motor symptoms of Parkinson’s disease.^6,19-21^ Also, beta-band LFPs show a high consistency over time, being recordable 7 years after STN implantation,^22,23^ with changes in beta power strictly related to movement performance several months after surgery.^24^

Nonetheless, it is debated that the degree of synchronization in the beta band correlates with the severity of some symptoms before treatment, suggesting that beta oscillations do not completely account for motor impairment.^25,26^ In addition, aDBS triggered by power beta oscillations does not significantly reduce either speech or axial disturbances over time.^27^ More importantly, there is only a small amount of data discussing neurophysiological differences between the hemispheres.^28^ Recently, one study revealed that the degree of STN–STN synchronization in the beta range (13–30 Hz) is associated with worse bradykinesia but not with tremor or rigidity.^29^

From a clinical perspective, understanding the role and the extent of involvement of each hemisphere is important to consider the possibility of triggering only one STN during DBS surgery, at least for some clinical or neurophysiological phenotypes, especially when adaptive approaches are adopted. Although a different clinical impairment of either side represents a pathognomonic feature in patients with Parkinson’s disease, the two hemispheres show both different vulnerabilities to nigrostriatal denervation and different compensatory responses,^30^ which seem partly independent of the motor onset side.^31^ A recent EEG study has shown that an asymmetry of frontal cortex beta activity linearly correlates with disease severity, whereas a lateralization of occipital alpha activity predicts levodopa response.^31^ In this study, we investigated the combined contribution of left and right STN oscillations in patients with Parkinson’s disease, by using a phase-amplitude coupling (PAC) approach, before and after dopaminergic stimulation, by comparing either STN low- (12–20 Hz) or high-beta (21–30 Hz) oscillations with low- (LF: 30–45 Hz), medium- (MF: 70–100 Hz) and high-frequency (HF: 260–360 Hz) bands.^32,33^ This approach has some technical advantages compared with power spectrum analysis, being less dependent on the signal-to-noise ratio; in addition, it overcomes the movement-related reduction of high-alpha/low-beta oscillations and the possibility that in some patients, beta oscillations cannot be easily recorded.^34-36^

## Materials and methods

### Patients

Twenty-four patients (12 females, 12 males) with Parkinson’s disease were included in the study (Table 1). An informed consent form was signed before the enrolment, and the study was approved by the local ethical committee (according to the Declaration of Helsinki); the data were derived from the same data set used for previous papers published by our group.^37^ Patients underwent functional neurosurgery for bilateral implantation of DBS electrodes in the STN. The average age was 56 years (range 48–61 years), with a disease duration of ∼10 years (range 7–16 years), a levodopa equivalent pre-surgery mean dose of 830 mg/day (500–1500) and an UPDRS-III (Unified Parkinson’s Disease Rating Scale III motor part)^38^ pre-surgery off-therapy of 29 (13.5–64). The patients had a predominantly rigid/akinetic phenotype. Each patient fulfilled inclusion criteria for DBS treatment.^39^ Briefly, the anatomical target was identified through pre-operative direct visualization using CT–MRI-based targeting,^40,41^ followed by intra-operative neurophysiology with microrecordings,^42,43^ intra-operative stimulation (i.e. through the exploratory electrode) and macrostimulation (i.e. through the implanted macroelectrode), and finally, post-operative neuroimaging for the confirmation of electrode position. The implanted electrode for DBS (Model 3389; Medtronic Inc., Minneapolis, MN, USA) was composed of four metal contacts, designated as 0–1–2-3 along a caudal-to-rostral direction.

**Table 1:** Demographic and clinical features of patients

Patient	Gender	Age (years)	Recorded side	Recording condition (Med-OFF, Med-ON)	L-DOPA equivalent before surgery (mg)	Dopamine agonist dose before surgery	Motor onset side
1	F	54	R	OFF, ON	1500	4	Right
2	F	69	R, L	OFF, ON OFF, ON	1377	3	Right
3	M	48	R, L	OFF, ON ON	1140	2.4	Right
4	F	55	R, L	OFF, OFF	1040	2	Right
5	F	64	R	OFF	1995	0	Left
6	M	52	R, L	OFF, OFF	2400	0	Right
7	F	53	R	OFF	900	0	Left
8	M	66	R, L	OFF, OFF	975	0.36	Left
9	F	61	R, L	OFF, OFF	925	3	Right
10	M	63	R, L	OFF, OFF	1260	1.56	Right
11	M	59	R, L	OFF, ON OFF, ON	1800	3	Left
12	F	59	R, L	OFF, OFF	1671	2.34	Right
13	F	59	R, L	OFF, ON OFF, ON	1400	0	Right
14	M	67	L	OFF, ON	1000	3.12	Left
15	F	39	L	OFF, ON	800	3	Left
16	F	70	R, L	OFF, ON OFF, ON	1200	1.8	Right
17	M	44	L	OFF, ON	1500	0	Left
18	F	70	R, L	OFF, ON	1010	3	Right
19	M	56	L	OFF, ON	2800	14	Left
20	M	38	R, L	OFF, OFF	3230	5.6	Right
21	M	67	R, L	OFF, OFF	825	2.4	Right
22	M	63	R, L	ON, ON	1292	0	Right
23	F	55	R, L	ON, ON	1250	3	Left
24	M	66	R, L	ON, ON	900	0.7	Left

F, female; M, male; R, right; L, left.

Two or 3 days after surgery, following 12 h of medication withdrawal, each session started with a baseline evaluation (medication ‘OFF’). The patients took their first morning medication afterward and were then assessed when the medication became effective (assuming a peak dose of ∼45–60 min after the medication intake). The evaluation of both ON and OFF states was confirmed by clinical evaluation performed by two experienced neurologists.

UPDRS-III scores, before and after levodopa, selection of participants, DBS target stereotactic coordinates and estimated STN length have been reported elsewhere in detail.^37^

### LFP recordings and power spectral analysis

Forty-one STN–LFPs (20 right and 21 left) were recorded from the 24 bilaterally implanted subjects included in the study and were analysed pre- and post-dopamine treatment (formally named as OFF and ON conditions). The row signals were pre-amplified, filtered (band pass 2–1000 Hz) and differentially amplified (100 000×.) with an analogical amplifier (Signal Conditioner Cambridge 1902; Cambridge Electronic Design, Cambridge, England). Signals were then recorded in four different combinations of pre- and post-levodopa with a mean dose of 830 mg/day (500–1500) administered before surgery on both sides: 17 STN no levodopa on the left (Med: OFF, Side: left), 16 no levodopa of the right (Med: OFF, Side: left), 13 levodopa on the left (Med: ON, Side: left) and 9 levodopa on the right (Med: ON, Side: right). The sampling frequency was set at 2500 Hz, and a 12-bit quantization with a 5 V range and a classical LFP pre-processing procedure was performed in order to reduce noise and signal variability.^14,17^ Therefore, a high pass filter at 2 Hz was applied, a notch filter was used to remove the electrical power interference, and a normalization procedure was done by subtracting the mean and dividing the result by the standard deviation of the filtered signals. On processed signals, time windows of 43 s were then selected, and the power spectral analysis of each segment of LFP was estimated using Welch’s method. A fast Fourier transform was computed at each side with a 1 s Hamming window and 50% overlap. For each LFP band, spectral powers were extracted and calculated, defined as the average power in a band expressed after decimal logarithmic transformation (log power) in low beta (12–20 Hz) and high beta (21–30 Hz), separately.^44^ For each patient, two 60 s-long epochs of LFP were extracted at each specific experimental phase (i.e. before and after levodopa). Data were divided into 15 overlapping and evenly distributed subepochs, each comprising 90% of the total length of the original epoch. Data were tapered with a Hanning window and for each subepoch. The spectral power was computed as follows:


(1) $$P_{\left(\;f_{1}-f_{2}\right)}=\frac{1}{\;f_{2}-f_{1}}\int_{\;f_{1}}^{\;f_{2}}\mathrm{PSD}(f)\;df$$

where $f_{1}$ and $f_{2}$ represent the boundary frequencies of the considered band $\left(\;f_{1}-f_{2}\right)$, $P(\;f_{1}-f_{2})$ is the spectral power in the band and PSD(f) is the spectral power at the frequency (f).^44^

### Phase-amplitude coupling

We used a cross-frequency measure, previously, to analyse frequency ranges for phase-to-amplitude modulation.^37^ PACs of high and low beta were evaluated in relation to three different ranges based on the literature of frequency described here^37,45^: LFs (30–45 Hz), MFs (70–100 Hz) and HFs (270–360 Hz). The phase-to-amplitude comodulograms were created using a modulation index (MI) measure, which was applied to various pairs of frequency bands: 1 Hz bin for the ‘phase frequency’ and a 5 Hz bin for the ‘amplitude frequency’ bands.^37^ The MI measure is centred on a normalized entropy measure that has been shown to detect multimodal phase distributions using the nested-frequency analysis algorithm, as described by He *et al*.^46^ and further developed by Hurtado *et al*.^47^ PAC was evaluated for each frequency pair on a 2D frequency space, using frequency bins with 1 Hz width (0.5 steps) centred at 3, 4,…, 40 Hz for phase extraction (*f*_p_ plotted on the *x*-axis) and 5 Hz width frequency bins centred at 3, 8,…, 398 Hz for amplitude extraction (*f*_A_ plotted on the *y*-axis). In summary, for each pair of frequencies *f*_p_ and *f*_A_, an LFP was filtered within the corresponding frequency bins |*f*_p_| and |*f*_A_| using a third-order symmetric Butterworth filter with 60 s windows and linear-trend removal. The instantaneous phase $\phi_{\left(f_{p}\right)}(t)$ and amplitude $A_{\left(\;f_{A}\right)}(t)$ time series were then extracted using the standard Hilbert transform. The sample-by-sample values of $\phi_{\left(f_{p}\right)}(t)$ were divided into 0.1*π* width intervals ranging from −*π* to *π*, and the corresponding $A_{\left(\;f_{A}\right)}(t)$ values were averaged for each phase bin. To evaluate the PAC, an inverted entropy measure *H* was applied to the average $A_{\left(\;f_{A}\right)}$ values for each phase bin $\phi_{\left(f_{p}\right)}(j)$, where *j* = 1, 2, …, 20:


(2) $$H=-\sum_{\;j=1}^{N}\;p_{j}\log(\;p_{j})$$

where *N* = 20 (i.e. number of bins) and $p_{j}$ is


(3) $$\frac{{\left\langle A_{\;f_{A}}\right\rangle }_{\phi_{\;f_{p}}}(j)}{\sum_{\;j=1}^{N}{\left\langle A_{\;f_{A}}\right\rangle }_{\phi_{\;f_{p}}}(j)}$$

The MI is obtained by normalizing *H* by the maximum possible entropy value ($H_{\max}=\log\;N$, where *N* = 20) as


(4) $$\mathrm{MI}=\frac{H_{\max}-H}{H_{\max}}$$

Thus, a low MI indicates a lack of phase-to-amplitude modulation, and therefore, larger MI values result from a stronger phase-to-amplitude modulation. To determine the statistical significance of the MI values, they were compared against a distribution of 44 shuffled time series, generated using a shuffling procedure that preserves the temporal structure of the original signal. A *Z*-score statistic for MI was calculated by comparing the original values against the means and standard deviation of the shuffled MI. All analyses were performed using MATLAB R2020a software, with an adapted version of the Matlab code from He *et al*.^46^ used for PAC analysis (Supplementary Matlab Program Distribution, BNestedfreq.m).

### Statistical analysis

The Shapiro–Wilk test defined the distributions as non-normal; thus, non-parametric statistics were used. The Kruskal–Wallis test was performed, comparing left and right STNs under ON and OFF medication conditions. In this way, significant values (*P* < 0.05) were identified by estimating the error variance for independent measures in individual bands. Finally, the possible influence of the motor onset side was explored by dividing the patients into two groups depending on the motor onset side. Statistical analyses were performed by using JASP 0.16.1.0.

## Results

### Power spectrum density

**Figure 1:**
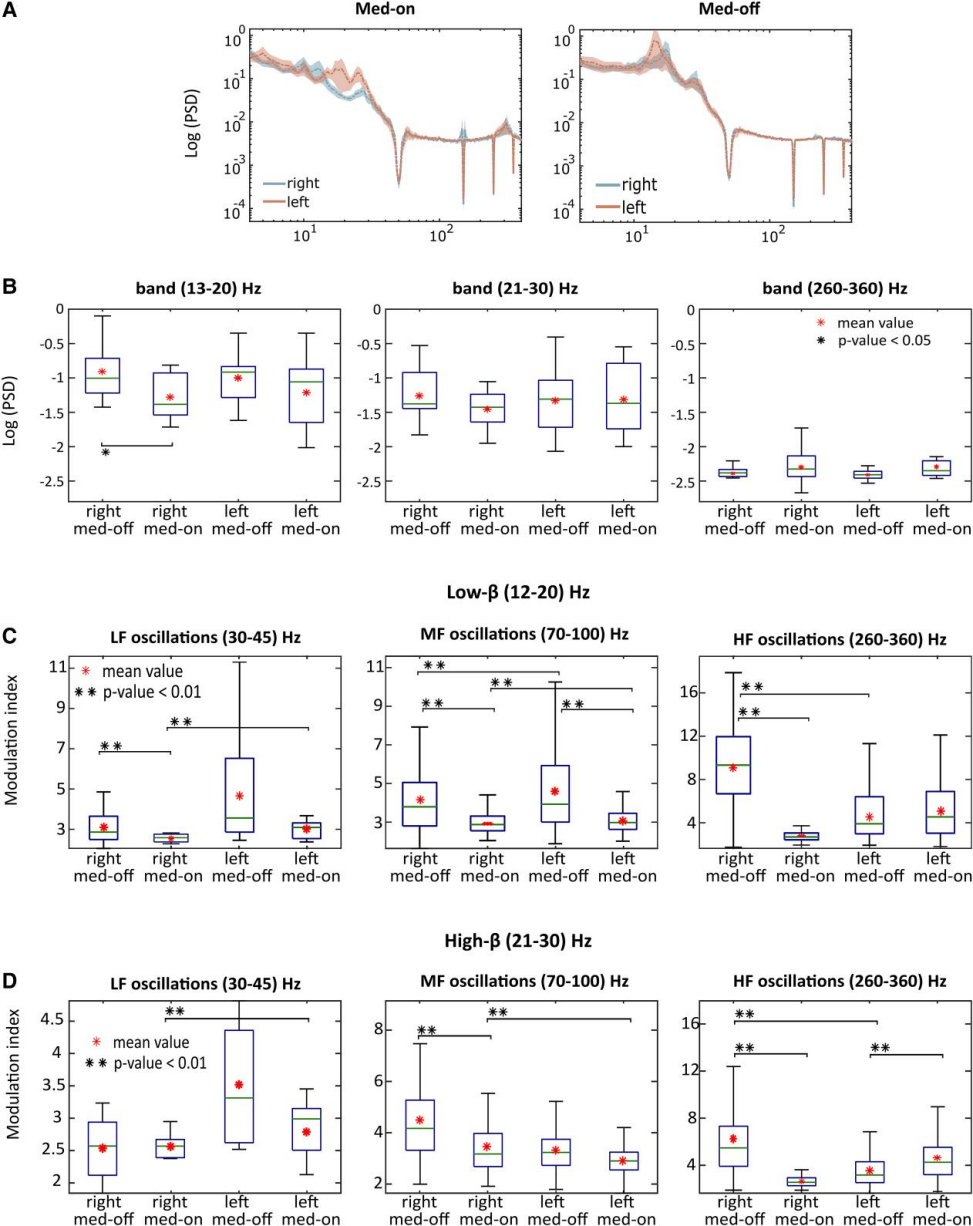
**A comparison of spectral power and PAC between left and right STNs during Med-OFF and Med-ON conditions.** A comparison of PSD and PAC between left and right STNs during medication OFF (Med-OFF) and medication ON (Med-ON) conditions. (**A**) Group-averaged (dotted line) PSDs and 95% confidence interval of the mean (shaded area) are plotted for both left and right STN during both Med-OFF (left) and Med-ON (right) conditions. (**B**) The box plots represent averaged PSD values in the LF, HF and HF bands between the two hemispheres and the two pharmacological treatments. Statistical differences are denoted with * (*P* < 0.05). (**C** and **D**) A box plot representing the averaged phase of coupling for both LF (**C**) and HF (**D**) bands between the two hemispheres and pharmacological treatments. Statistical differences are denoted with ** (*P* < 0.01, Kruskal–Wallis).

As reported in Fig. 1A and B, power spectrum density (PSD) revealed significant differences between the Med-OFF and the Med-ON conditions in the low-beta band in the right STN (right ‘logarithmic arbitrary units’, or log AU, ± std, Med-OFF −0.91 ± 0.47 versus Med-ON −1.33 ± 0.29; *P* = 0.004) but no difference in the left STN, i.e. between right and left STNs both in Med-OFF and Med-ON conditions. Also, PSD was similar between sides and medication conditions in the high-beta bands and other considered frequency bands.

### Phase-amplitude coupling

Differences between left and right STNs and between high- and low-beta PACs to LF, MF and HF in conditions of ON and OFF dopamine treatment are shown in Fig. 1C. Low- (12–20 Hz) and high-beta (21–30 Hz) ranges were used for phase extraction (*f*_p_ plotted on the *x*-axis, Fig. 1C and D) and provided statistically significant differences among right and left PACs in both Med-ON and Med-OFF conditions to 70–100 Hz (MF; Fig. 1) and 260–360 Hz (HF) used for amplitude extraction (*f*_A_ plotted on the *y*-axis; Fig. 1); no differences were found at 30–45 Hz (LF), except for the low-beta Med-OFF condition.

**Figure 2:**
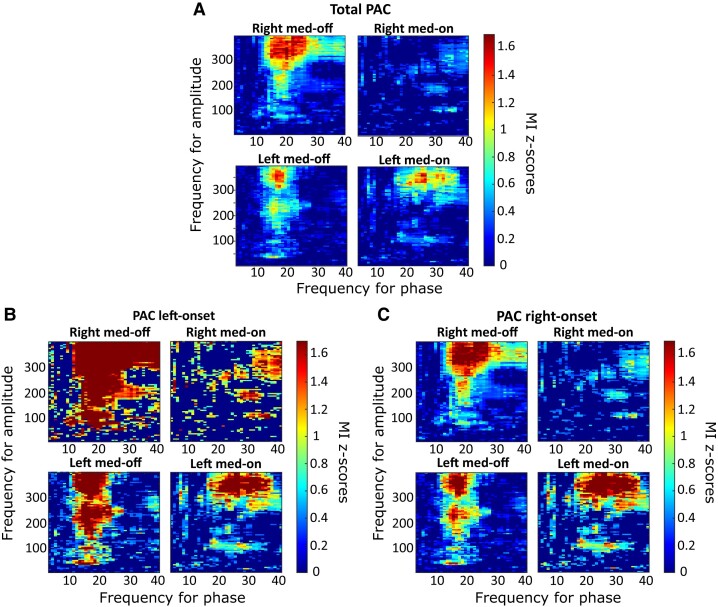
**Gross analysis of PAC.** (**A**) Average *Z*-score Bonferroni-corrected maps for the entire data set (16 Med-OFF right, 17 Med-OFF left, 9 Med-ON right, 13 Med-ON left nuclei) are represented for both left and right hemispheres during both Med-OFF and Med-ON. The phases that are considered here and represented on the horizontal axis range from 10 to 40; the coupled amplitude instead, represented on the vertical axis, ranges from 30 to 460 Hz. Warmer colours are indicative of a high PAC. (**B**) Average *Z*-score Bonferroni-corrected maps are presented for both left and right hemispheres, during Med-OFF and Med-ON conditions, in patients with a left onset of motor symptoms. (**C**) Average *Z*-score Bonferroni-corrected maps are presented for both left and right hemispheres, during Med-OFF and Med-ON conditions, in patients with a right onset of motor symptoms. When HF amplitudes were compared with high-beta phases, the right STN showed a higher coupling than the left during the OFF condition (*P* < 0.0005, Kruskal–Wallis), followed by a higher one during the ON state (*P* < 0.0001). Colour scales for the MI *Z*-score are reported at the right of each heat map.

**Figure 3:**
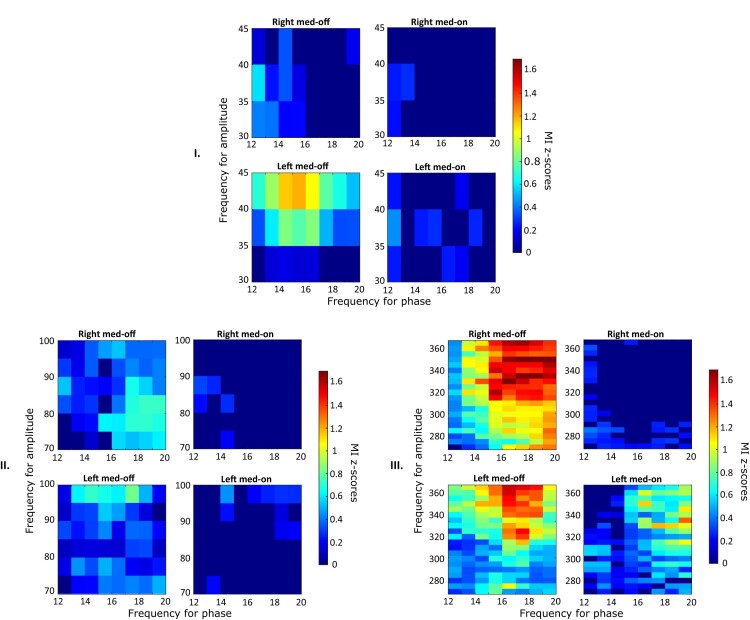
**Gross analysis and effect on PAC between low-beta frequencies and LF, MF and HF.** Average *Z*-score Bonferroni-corrected maps for the entire data set are represented for both left and right hemispheres during both Med-OFF and Med-ON. The phases that are considered here and represented on the horizontal axis range from 12 to 20; the coupled amplitude instead is in the range of each frequency band (Part I: LF; Part II: MF; Part III: HF).

In the left STN, the high beta coupled to MF (Fig. 2A) showed a lower MI in the Med-OFF condition (*P* < 0.01) and a higher one following levodopa intake (Med-ON: *P* < 0.01) when compared with the right STN. Also, when both low beta and high beta were considered together for the analysis, the left STN showed a lower MI in the Med-OFF condition, both for LF (*P* < 0.01) and MF couplings (*P* = 0.006), and a higher MI following levodopa intake, compared with the contralateral one (coupled to HF: *P* < 0.001). However, when data were analysed in terms of PAC between low-beta and LF or MF oscillations, depending on the motor onset side (either right or left), there were no differences between the two sides, before and after levodopa intake (Fig. 3, *P* > 0.1).

**Figure 4:**
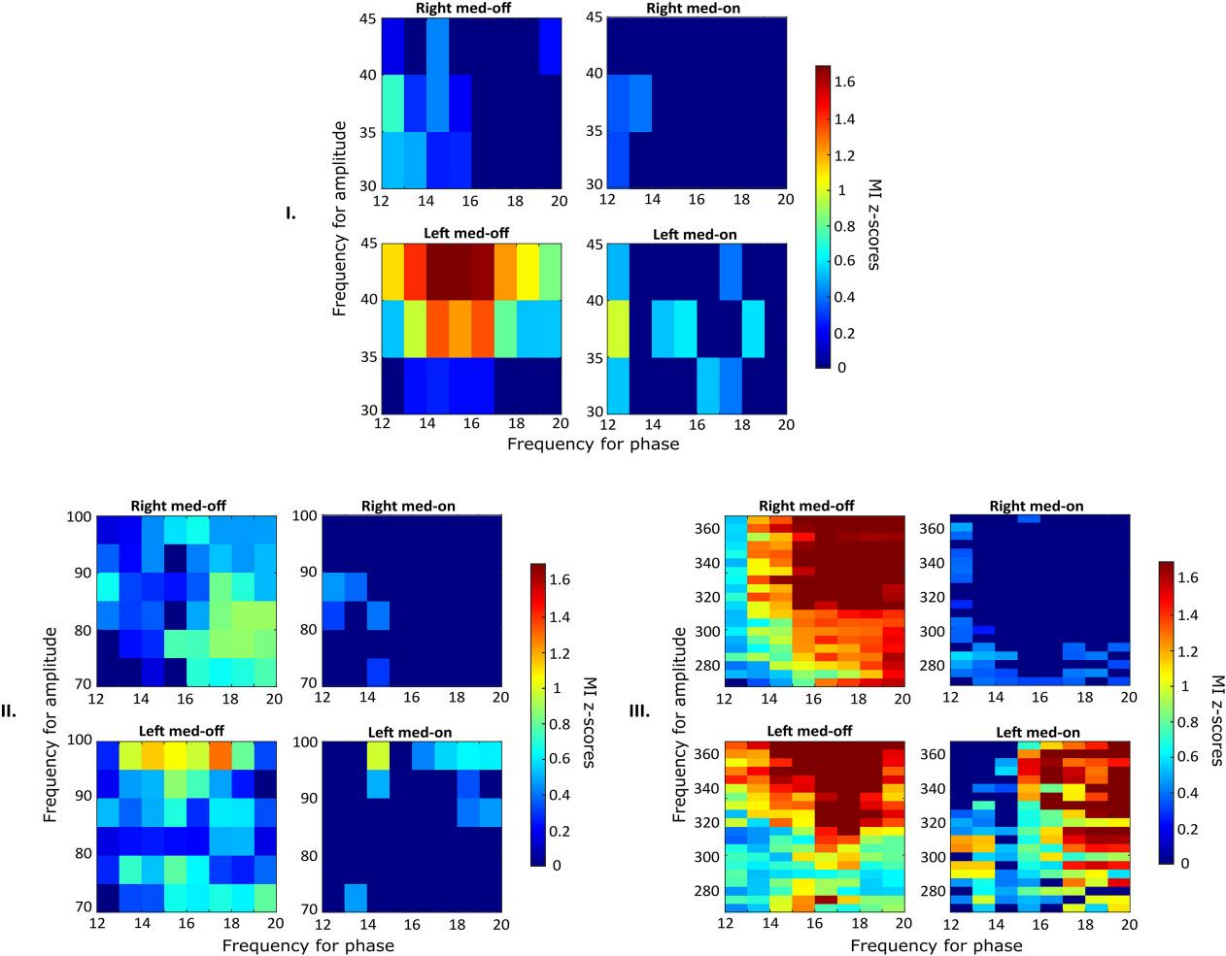
**Gross analysis and effect of the motor onset side on PAC between low-beta frequencies and LF, MF and HF (right-side onset).** The figure represents the analysis when the motor onset side was evaluated separately (i.e. for Parkinson’s disease with a right onset). The phases that are considered here and represented on the horizontal axis range from 12 to 20; the coupled amplitude instead is in the range of each frequency band (Part I: LF; Part II: MF; Part III: HF). Colour scales for the MI *Z*-score are reported at the right of each heat map.

**Figure 5:**
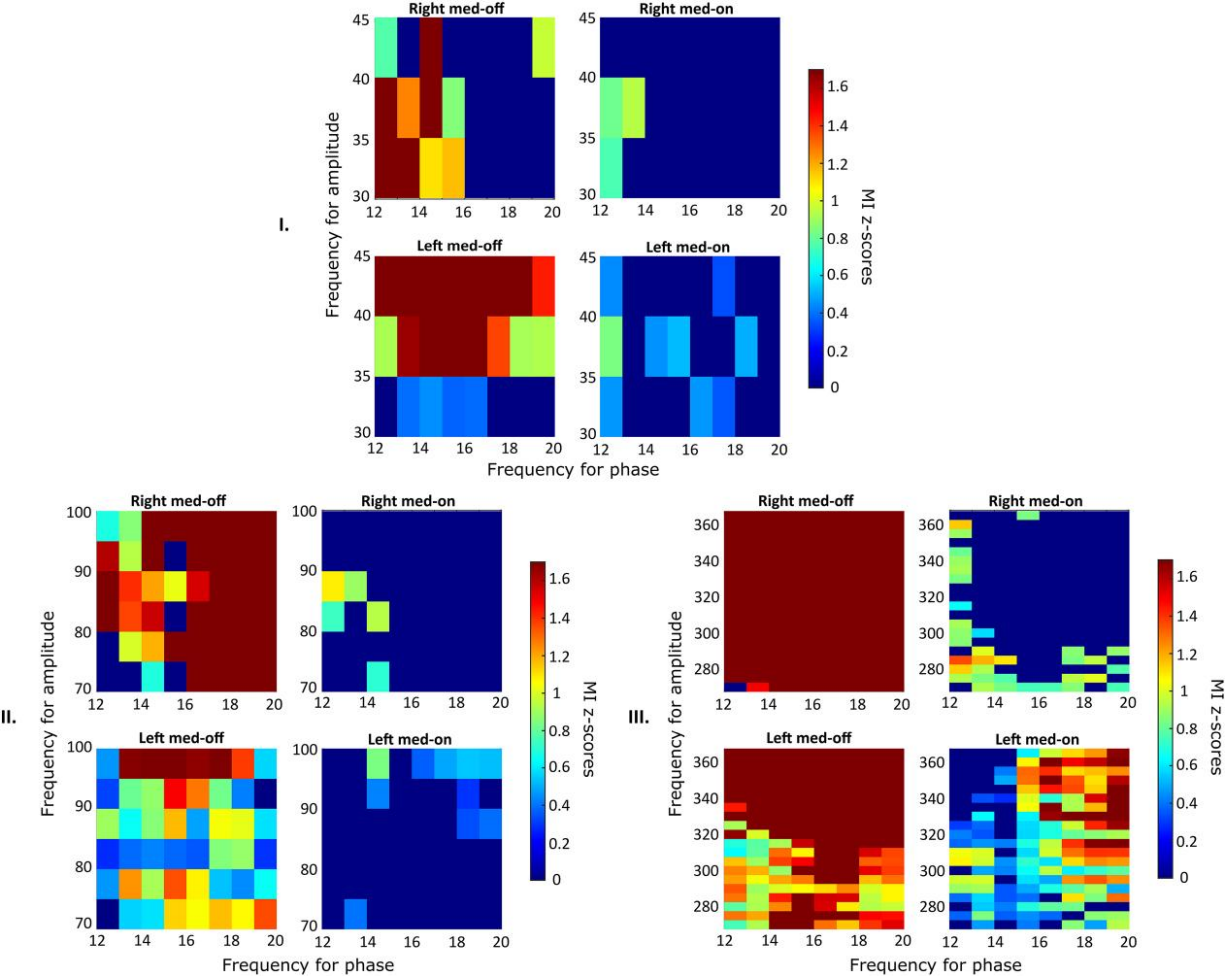
**Gross analysis and effect of the motor onset side on PAC between low-beta frequencies and LF, MF and HF (left-side onset).** The figure represents the analysis when the motor onset side was evaluated separately (i.e. for Parkinson’s disease with a left onset). The phases that are considered here and represented on the horizontal axis range from 12 to 20; the coupled amplitude instead is in the range of each frequency band (Part I: LF; Part II: MF; Part III: HF). Of note, a higher MI was identified in the right STN compared with the contralateral one (*P* < 0.001). Colour scales for the MI *Z*-score are reported at the right of each heat map.

**Figure 6:**
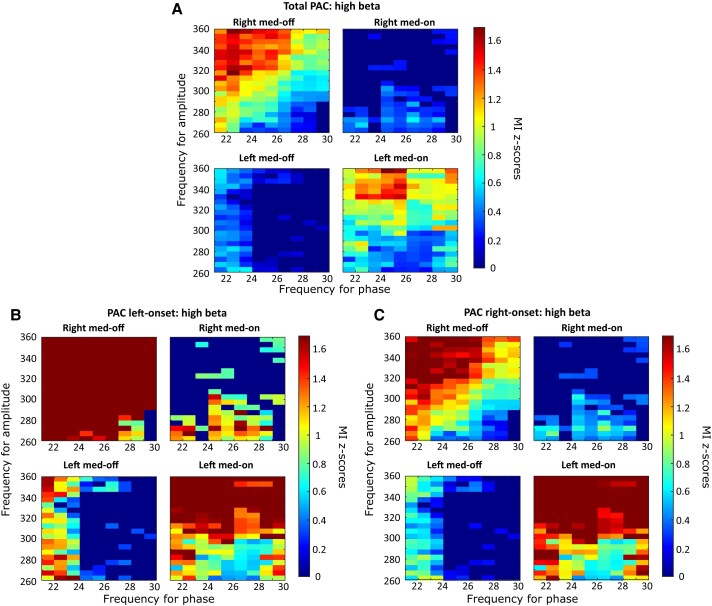
**Effect of the motor onset side on PAC between 22–30 Hz and HF.** (**A**) Average *Z*-score Bonferroni-corrected maps for the entire data set (16 Med-OFF right, 17 Med-OFF left, 9 Med-ON right, 13 Med-ON left nuclei) are represented for both left and right hemispheres during both Med-OFF and Med-ON. The phases that are considered here and represented on the horizontal axis range from 22 to 30; the coupled amplitude instead, represented on the vertical axis, is in the range of the HF band (260–360 Hz). Warmer colours are indicative of a high PAC. (**B**) Maps relative to the patients having a left-side onset disease. (**C**) Maps relative to the patients having a right-side onset disease. Colour scales for the MI *Z*-score are reported at the right of each heat map.

Finally, the patients were divided into two groups, depending on motor onset side (left versus right). We first assessed inter-hemispheric differences between a wide spectrum of frequencies (LF, MF, HF amplitudes and low- and high-beta phases, Figs 4–6, respectively); then, we analysed differences between HF amplitudes and either high- or low-beta-band phases. First, differences were found by comparing the two hemispheres in terms of PAC between LB and HF after levodopa intake; a higher MI was identified in the left STN compared with the contralateral one (*P* < 0.001; Figs 4 and 5), which was independent of the motor onset side (*P* = 0.21; Figs 4 and 5). Second, the coupling between MF–HF and high-beta oscillations showed a higher MI during the OFF condition (*P* < 0.0001) and a lower coupling during the ON state (*P* < 0.0001) in the right STN, independent of the motor onset side (Fig. 2B and C). More specifically, as graphically reported in Fig. 6, inter-hemispheric differences are particularly evident when HF amplitudes were compared with high-beta phases, with the right STN showing a higher coupling than the left during the OFF condition (*P* < 0.0005), followed by a higher one during the ON state (*P* < 0.0001), which is independent of the motor onset side.

## Discussion

In our study, we investigated changes in PAC, before and after levodopa administration, by comparing right and left STN and low- or high-beta oscillations with LF (30–45 Hz), MF (70–100 Hz) and HF (260–360 Hz) bands. Our data confirmed that the two sides of the brain are distinctly involved in Parkinson’s disease, supporting the notion that the lateralization of different frequency bands provides different clues underlying Parkinson’s disease pathophysiology. Here, we used the method of PAC in order to compare different frequency oscillations, possibly arising from different STN neuronal subpopulations with distinct functions within the basal ganglia network. Although this approach is commonly used to compare languages among different nuclei or cortical areas, it has also been adopted for the evaluation of intra-subthalamic oscillations.^36^ The changes we observed for low-beta oscillations are in line with the well-known role of STN beta frequencies as an electrophysiological marker of the disease: their amplitude decreases during the ON state, paralleled by a facilitation of gamma frequencies, which are considered ‘pro-kinetic’, resulting in an overall phase-amplitude decoupling.^48-51^ Nonetheless, a peculiar trend was observed when high-beta frequencies and either MFs or HFs were compared and, partially, between low-beta frequencies and HFs. Indeed, PAC was reduced by recording from the right STN, as expected, whereas a significant increase during the ON state was found when analysing the signals derived from the left side. More importantly, these changes did not correlate with the onset side, either left or right. However, due to missing data and the limited sample size, it was not possible to establish a clear relationship between this asymmetry and manual dexterity.

Particularly, the involvement of MFs (70–100 Hz) seems to support previous papers showing a dopamine-dependent and finely tuned scaling of movement-related synchronization within the range of gamma frequencies and highlighting the role of this band in the control of bradykinesia and axial symptoms in Parkinson’s disease.^52-54^ Moreover, it has been recently suggested that effective HF STN–DBS normalizes the balance between beta and gamma oscillations at a cortico-subcortical level.^54^

More importantly, and to the best of our knowledge, no previous study has assessed the neurophysiological hemispheric asymmetry in Parkinson’s disease by evaluating subthalamic LFPs. Although their significance and the underlying pathophysiological bases are still to be elucidated, our findings argue against a randomly asymmetric vulnerability of STN dopaminergic neurons. Only one paper adopted the same approach to study PAC, revealing a strong correlation with the more affected hemisphere, without evaluating the effects of dopaminergic stimulation.^36^ The changes that we observed in the right versus left STN may be explained, at least in part, by the lateralization of the hyper-direct pathway (HDP) of the basal ganglia network.^55^ This pathway involves the right hemisphere and exerts an overall inhibitory role on the thalamo-cortical output.^55^ A surgery of the right STN may functionally disrupt this pathway, thus resulting in a more pronounced decoupling between ‘bradykinetic’ beta and ‘pro-kinetic’ gamma frequencies. This possibility is strongly supported by the notion that high-beta oscillations are now considered the neurophysiological hallmark of the HDP.^56,57^

Another possibility may involve the role of the right STN on axial motor symptoms.^24,58^ Usually, the right STN exerts an inhibitory control over the left one on axial motor control^59-61^; accordingly, patients with freezing of gait (FOG) show abnormally reduced structural connectivity on diffusion tensor imaging and functional MRI preferentially affecting right motor circuits during gait imagery tasks.^62-64^ These findings are further confirmed by the observation that unilateral STN–DBS alleviates axial symptoms and FOG to a greater extent compared with either bilateral STN–DBS or dopaminergic stimulation.^65^

A third hypothesis may consider the origin of beta oscillations in Parkinson’s disease. In particular, a previous paper showed that dopaminergic stimulation does not alter STN–STN coherence, inducing the beta-band activity to switch from an STN-mediated motor network to a frontoparietal-mediated one.^65^ Although the authors adopted a different protocol than ours and assessed STN oscillations until the beta range without assessing HF bands, their results lead to a consideration of the asymmetry that we described here not as a dysfunctional feature intrinsic to the basal ganglia network. In this context, we can also explain the significant changes that we observed when PAC between low-beta and HF oscillations was analysed, as a broad perturbation of networks.

High-beta oscillations are thought to express inter-regional, rather than local, brain dynamics, predicting the transition from one clinical state to the other earlier than low-beta frequencies.^57,66^ This may explain the inter-hemispheric asymmetries that we found only when high-beta oscillations and HFs were compared, as well as the differences in high-beta and HF coupling between hemispheres during the OFF phase.

Previous papers have reported higher levels of dopamine in the left versus right basal ganglia, suggesting that the right hemisphere is more vulnerable to dopamine depletion, independent of the side onset of the disease.^67-70^ Accordingly, others reported slightly higher concentrations of [^18^F]-DOPA and [^123^I]b-CIT tracer binding in the left striatum of healthy volunteers.^71,72^ This predominance has been postulated to play a major role in motor lateralization and in the acquisition of bimanual movements in humans.^73,74^ A post-mortem study on brains not affected by neurodegenerative disorders confirmed higher dopamine levels in the left compared with the right striatum.^75^ Other data have recently described a left-predominant susceptibility to neurodegeneration, both at a cortical^76^ and at a subcortical level.^77^ Scherfler *et al*.^78^ showed a left hemispheric predominance of nigrostriatal dysfunction by analysing putaminal dopamine transporter availability, confirming previous clinical observations of a greater proportion of right-handed patients with Parkinson’s disease with predominantly right-sided motor signs.^79,80^ Nonetheless, these results do not completely explain the considerable number of right-handed patients with Parkinson’s disease with left disease predominance and with lower dopamine transporter binding of their right putamen.^78,81^

### Limitations

This study presents some limitations. First, the duration of LFPs recordings was sometimes too short (sometimes <1 min), partly due to intra-operative concerns. Second, data about hand dominance, possibly interfering with disease onset and the activation of compensatory mechanisms, in many patients were lacking and did not allow further analyses.

A third possible limitation is that different PAC patterns between hemispheres may reflect the asymmetry in the severity of motor symptoms rather than the asymmetry in the anatomical side. Moreover, the asymmetry between the more and less affected striatum becomes less prominent as the disease progresses, in terms of dopamine synthesis, storage and reuptake.^82^ However, although there is a tendency towards the development of a more symmetric distribution of motor features with increasing age, it does not represent an independent factor predicting disease severity over time^83^; the motor distribution pattern seems to remain stable and continues to correlate with a reduction in dopamine tracer binding,^84^ especially in the caudate and anterior putamen.

## Conclusion

The lateralization of cortical/subcortical activity may predict response to treatment and highlight novel insights into the pathophysiology of Parkinson’s disease. Particularly, the results presented here may raise questions about the use of new electrophysiological markers for guiding adaptive DBS approaches. For instance, inter-hemispheric coupling in the beta range, but not power spectrum or burst dynamics, seems to correlate with progressive worsening in bradykinesia over time.^29,58^

In addition, these data may suggest the possibility of applying the surgical procedure to one hemisphere only, possibly the right one, at least in a few selected patients. This could reduce surgical times and perioperative complications, preserving battery life. In particular, this opportunity is supported by the finding that patients with a higher coupling between high beta (22–30 Hz) and HFs (300–400 Hz) during the ON state showed a significantly greater reduction in the symptoms of bradykinesia.^57^

Further studies are needed to prove the correlation between PAC and motor onset, as well as between PAC and response to levodopa administration, possibly recording neuronal oscillations from different brain regions in order to validate these measure as a promising neurophysiological marker (e.g. between the motor cortex and STN or the internal ‘globus pallidus’, GPi).

## Data Availability

The data that support the findings of this study will be available from the corresponding author on request.
